# Trends and disparities in retention to antiretroviral therapy of people living with HIV from 2014 to 2022 in Brazil: a population-based study

**DOI:** 10.1016/j.bjid.2026.105804

**Published:** 2026-03-30

**Authors:** Marcelo A. de Freitas, Rosana E.G.G. Pinho, Ana Roberta P. Pascom, Angelica E. Miranda

**Affiliations:** a Universidade Federal do Espírito Santo, Programa de Pós-Graduação em Doenças Infecciosas, Vitoria, ES, Brazil; b Ministério da Saúde, Brasília, DF, Brazil

**Keywords:** HIV, AIDS, Retention, Antiretroviral therapy

## Abstract

**Introduction:**

Retention to antiretroviral therapy (ART) is critical for controlling HIV/AIDS and reducing mortality rates worldwide. In Brazil, although ART is widely accessible, challenges remain in retaining individuals on treatment over time. This study aims to analyze trends in ART retention from 2014 to 2022, addressing gaps in current national data.

**Methods:**

This is a population-based, cross-sectional study, that utilized data from the Brazilian national ART monitoring system to assess retention rates at 6-, 12-, 24-, 36-, 48-, and 60-months post-ART initiation. The analysis included individuals aged 15-years or older, with retention defined as no more than a 28-day delay in drug distribution. Descriptive analyses of retention from 2014 to 2022 were conducted using R version 4.0 (R Core Team, 2020) and SPSS version 21 (IBM Corp.)

**Results:**

The study observed over 3.4 million ART distributions, revealing that retention rates steadily declined over time, with the lowest rates observed at 60-months (71% in 2022). Men had consistently higher retention rates compared to women (79% vs. 73% in 2022). Lower retention was found among younger age groups (74% in 2022), indigenous populations (67% in 2022), individuals with lower education levels (72% in 2022), and those residing in northern (74% in 2022) regions of Brazil.

**Conclusions:**

The study highlights considerable retention challenges, particularly after extended periods on ART, with notable disparities across demographic and regional groups. The findings suggest the need of interventions to improve ART retention, especially among vulnerable populations, to reach the 95–95–95 targets in Brazil. The introduction of multi-month drug dispensing and other strategies implemented during the COVID-19 pandemic may have helped stabilize retention rates in recent years.

## Introduction

Antiretroviral Therapy (ART) has transformed the landscape of HIV treatment, significantly reducing mortality and improving the quality of life for people living with HIV/AIDS (PLWHA) worldwide. The introduction of ART marked a pivotal moment in the global fight against HIV, offering hope not only for individual survival but also for broader public health goals, such as viral suppression and epidemic control.^1^^,^^2^ However, the full potential of ART can only be realized if individuals are consistently retained in treatment.^3^, ^4^, ^5^ Retention in care ensures long-term viral suppression, prevents disease progression, and reduces transmission, yet achieving high retention rates remains a persistent challenge across various regions and demographic groups.^6^, ^7^, ^8^, ^9^, ^10^, ^11^

In low- and middle-income countries, where the burden of HIV is disproportionately high, retention to ART is critically influenced by socioeconomic, demographic, and systemic factors. A 2016 meta-analysis estimated retention rates at 12-, 24-, and 36-months in these countries, revealing stark regional disparities.^12^ While these trends highlight the importance of sustained ART engagement, country-specific analyses are essential to uncover local barriers and tailor effective interventions, particularly in settings like Brazil, which has a longstanding universal ART policy but still faces unique implementation challenges.

Although ART retention in Brazil has been reported in the past by Ministry of Health, it has not fully captured the nuances of treatment retention over time, particularly considering the growing adoption of multi-month dispensing strategies.^13^ Furthermore, limited research has explored how ART retention varies by demographic factors such as age, sex, race, education, and region, leaving significant gaps in our understanding of retention dynamics in the country.

Understanding trends and disparities in retention to Antiretroviral Therapy (ART) across different sociodemographic groups is crucial for informing public health strategies. The findings of this study can support the Brazilian Ministry of Health and other stakeholders in designing and implementing more effective, equity-focused interventions. By identifying populations at greater risk of disengagement from care, such as specific age groups, racial/ethnic communities, or geographic regions, targeted policies and tailored retention strategies can be developed to improve outcomes and advance progress toward national and global HIV targets. Therefore, this study aims to analyze the trends in ART retention from 2014 to 2022 in Brazil, addressing gaps in current national data.

## Methods

### Study design and data source

This is a population-based, cross-sectional study, using secondary data from the Sistema de Controle Logístico de Medicamentos (SICLOM), the national information system for antiretroviral (ARV) dispensation in Brazil, which is an electronic, web-based platform that enables real-time registration and monitoring of information related to the distribution of medications. This includes patient registration, inventory control at health facilities, and the generation of reports to support the management and planning of public health actions. SICLOM stores data on all PLWHA who receive ART in the country. ARV dispensation is conditional upon the mandatory input of patient data into the system, making SICLOM a comprehensive source of treatment data.^14^

Because all ARVs for HIV treatment are provided exclusively by the public health system, regardless of whether patients are followed in public or private services and are not available for purchase outside this system (e.g., pharmacies), SICLOM captures information on the entire population of PLWHA on ART in Brazil. Additionally, SICLOM tracks dispensations nationally, ensuring continuity of data even when patients transfer between regions.

### Study population

No sample size was defined, as the study included all PLWHA aged 15-years or older who had at least one ART dispensation recorded in SICLOM between January 1, 2014, and December 31, 2022. This comprehensive inclusion ensured national representativeness.

### ART retention assessment

ART retention was evaluated at six time points after ART initiation: 6-, 12-, 24-, 36-, 48-, and 60-months. For each individual and each year of the analysis period, we determined the exact date corresponding to these intervals from ART initiation.

Retention status was determined by analyzing the timing of the most recent ARV dispensation prior to each time point. The interval between this dispensation and the target date was calculated. Information on the type of dispensation, whether monthly or multi-month (e.g., 30-, 60-, or 90-days), was considered, as this is recorded in SICLOM.

Following the Global AIDS Monitoring (GAM) framework, individuals were considered retained on ART if they had no delay or a delay of 28-days or less in their ART pickup at the specified time points (6-, 12-, 24-, 36-, 48-, or 60-months after ART initiation).^15^

### Data analysis

Data were organized by duration since ART initiation (at each time interval) and stratified by sex, race/color, age, region, and level of education. Descriptive trend analyses were conducted to explore retention patterns over time and across population subgroups. Available data were aggregated data on the number of patients using ART each year. No nominal data were used in the analysis.

The study used aggregated data on the annual number of patients receiving antiretroviral therapy (ART), without any individual-level (nominal) identification. All reported cases were included in the analysis; however, because the data are not linked at the individual level, it was not possible to determine whether the same patients were represented across multiple years. The data used are publicly available at: https://www.gov.br/aids/pt-br/indicadores-epidemiologicos/painel-de-monitoramento/painel-integrado-de-monitoramento-do-cuidado-do-hiv. Cases with missing information were classified as “unknown”.

A descriptive time trend analysis was conducted, stratified by sex, race/skin color, age, geographic region, and years of schooling. Descriptive analyses of retention from 2014 to 2022 were conducted using R version 4.0 (R Core Team, 2020) and SPSS version 21 (IBM Corp.). The data extraction for this study occurred between April 1 and April 7, 2023.

### Ethical considerations

This study was approved by the Ethics Committee of the Federal University of Espírito Santo (Approval n° 4.187.656/2020), with authorization from the Brazilian Ministry of Health. The requirement for informed consent was waived due to the use of anonymized secondary data. Data confidentiality was ensured through anonymization and the generation of unique personal identifiers. Access to identifiable data and the anonymization process were restricted to one author, also the designated data analyst and statistician responsible for managing the SICLOM database within the Ministry of Health.

## Results

A total of 3404,469 ARV dispensations were analyzed, with similar distribution among the different cut-offs of time from ART initiation analyzed: 6-months (18 %), 12-, 24- and 36-months (17 % each), 48-months (16 %) and 60-months (15 %). Most of the dispensations analyzed were made to male (68 %), black individuals (43 %), with a predominance of people aged 30- to 49-years (54 %). Regarding the region, 42 % of the ARV dispensations analyzed occurred in the Southeast region, followed by the Northeast (21 %) and South (20 %) regions. In addition, 23 % of ARV dispensations were done to individuals with 8- to 11-years of schooling, followed by 0- to 7-years (22 %) and 12 and more years (17 %). The proportion of missing data was <1 % for all variables, except for race/color (19 %) and education (39 %). The characteristics of PLWHA whose ARV dispensations were analyzed in our study are presented in Table 1.

**Table 1:** Characteristics of PLWHA whose ARV dispensations were analyzed in the study, according to time of ART initiation, sex, race/color, age, region and schooling. Brazil, 2014 to 2022.

**Characteristics**		**2014**		**2015**		**2016**		**2017**		**2018**	
		**n**	**%**	**n**	**%**	**n**	**%**	**n**	**%**	**n**	**%**
Time after ART initiation	6 months	65,479	22 %	71,032	21 %	71,059	19 %	67,640	17 %	69,910	17 %
	12 months	55,778	18 %	71,550	21 %	73,108	20 %	67,776	18 %	70,046	17 %
	24 months	51,466	17 %	55,778	17 %	71,558	20 %	73,100	19 %	67,776	17 %
	36 months	42,482	14 %	51,466	15 %	55,790	15 %	71,546	18 %	73,100	18 %
	48 months	43,766	14 %	42,482	13 %	51,471	14 %	55,785	14 %	71,546	18 %
	60 months	44,977	15 %	43,766	13 %	42,566	12 %	51,387	13 %	55,785	14 %
Sex	Male	187,280	62 %	214,091	64 %	239,690	66 %	260,176	67 %	281,096	69 %
	Female	116,666	38 %	121,980	36 %	125,858	34 %	127,055	33 %	127,063	31 %
	Missing	2	0 %	3	0 %	4	0 %	3	0 %	4	0 %
Race/color	White/yellow	124,912	41 %	135,812	40 %	145,122	40 %	150,433	39 %	156,365	38 %
	Black	113,837	37 %	131,251	39 %	148,837	41 %	162,092	42 %	175,710	43 %
	Indigenous	500	0 %	551	0 %	595	0 %	688	0 %	743	0 %
	Missing	64,699	21 %	68,460	20 %	70,998	19 %	74,021	19 %	75,345	18 %
Age	15 to 17 y.o.	1792	1 %	1986	1 %	2136	1 %	1998	1 %	1807	0 %
	18 to 24 y.o.	21,136	7 %	28,060	8 %	34,840	10 %	39,407	10 %	43,499	11 %
	25 to 29 y.o.	37,444	12 %	45,771	14 %	53,437	15 %	59,169	15 %	66,257	16 %
	30 to 49 y.o.	178,862	59 %	192,301	57 %	203,696	56 %	212,285	55 %	220,662	54 %
	50+ *y*.o.	59,989	20 %	63,576	19 %	67,272	18 %	70,462	18 %	72,217	18 %
	Missing	4725	2 %	4380	1 %	4171	1 %	3913	1 %	3721	1 %
Region	North	24,645	8 %	29,845	9 %	35,699	10 %	39,237	10 %	39,068	10 %
	Northeast	55,152	18 %	63,261	19 %	69,593	19 %	75,586	20 %	83,513	20 %
	Southeast	138,892	46 %	147,907	44 %	157,255	43 %	163,971	42 %	171,267	42 %
	South	64,548	21 %	71,552	21 %	77,670	21 %	80,607	21 %	83,972	21 %
	Mid-West	19,753	6 %	22,614	7 %	24,430	7 %	26,853	7 %	29,322	7 %
	Missing	958	0 %	895	0 %	905	0 %	980	0 %	1021	0 %
Schooling	0 to 7 years	77,397	25 %	81,247	24 %	83,458	23 %	86,089	22 %	87,557	21 %
	8 to 11 years	63,207	21 %	71,266	21 %	79,282	22 %	86,664	22 %	92,528	23 %
	12+ years	40,604	13 %	47,840	14 %	54,870	15 %	60,690	16 %	66,314	16 %
	Missing	122,740	40 %	135,721	40 %	147,942	40 %	153,791	40 %	161,764	40 %
**Characteristics**		**2019**		**2020**		**2021**		**2022**		**Total**	
		**n**	**%**	**n**	**%**	**n**	**%**	**n**	**%**	**n**	**%**
Time after ART initiation	6 months	68,785	16 %	61,925	15 %	56,053	15 %	63,970	16 %	595,853	18 %
	12 months	68,558	16 %	68,349	17 %	55,129	14 %	61,958	16 %	592,252	17 %
	24 months	70,046	17 %	68,564	17 %	68,343	18 %	55,129	14 %	581,760	17 %
	36 months	67,776	16 %	70,052	17 %	68,558	18 %	68,343	18 %	569,113	17 %
	48 months	73,100	17 %	67,782	17 %	70,046	18 %	68,558	18 %	544,536	16 %
	60 months	71,546	17 %	73,116	18 %	67,766	18 %	70,046	18 %	520,955	15 %
Sex	Male	294,696	70 %	292,455	71 %	278,985	72 %	283,075	73 %	2331,544	68 %
	Female	125,110	30 %	117,324	29 %	106,897	28 %	104,909	27 %	1072,862	32 %
	Missing	5	0 %	9	0 %	13	0 %	20	0 %	63	0 %
Race/color	White/yellow	156,088	37 %	148,797	36 %	135,865	35 %	132,956	34 %	1286,350	38 %
	Black	187,149	45 %	189,359	46 %	183,237	47 %	189,461	49 %	1480,933	43 %
	Indigenous	736	0 %	734	0 %	705	0 %	701	0 %	5953	0 %
	Missing	75,838	18 %	70,898	17 %	66,088	17 %	64,886	17 %	631,233	19 %
Age	15 to 17 y.o.	1779	0 %	1650	0 %	1425	0 %	1391	0 %	15,964	0 %
	18 to 24 y.o.	45,984	11 %	45,197	11 %	42,127	11 %	41,939	11 %	342,189	10 %
	25 to 29 y.o.	70,793	17 %	72,466	18 %	71,365	18 %	73,418	19 %	550,120	16 %
	30 to 49 y.o.	224,140	53 %	216,335	53 %	202,594	52 %	202,271	52 %	1853,146	54 %
	50+ *y*.o.	73,729	18 %	71,219	17 %	65,833	17 %	66,590	17 %	610,887	18 %
	Missing	3386	1 %	2921	1 %	2551	1 %	2395	1 %	32,163	1 %
Region	North	41,440	10 %	41,576	10 %	40,539	11 %	42,225	11 %	334,274	10 %
	Northeast	89,553	21 %	90,226	22 %	86,513	22 %	89,239	23 %	702,636	21 %
	Southeast	173,363	41 %	165,221	40 %	153,445	40 %	151,813	39 %	1423,134	42 %
	South	83,732	20 %	80,187	20 %	73,440	19 %	72,250	19 %	687,958	20 %
	Mid-West	30,622	7 %	31,237	8 %	30,606	8 %	31,213	8 %	246,650	7 %
	Missing	1101	0 %	1341	0 %	1352	0 %	1264	0 %	9817	0 %
Schooling	0 to 7 years	87,773	21 %	83,084	20 %	77,399	20 %	75,906	20 %	739,910	22 %
	8 to 11 years	97,209	23 %	98,644	24 %	95,956	25 %	98,684	25 %	783,440	23 %
	12+ years	69,987	17 %	70,652	17 %	73,065	19 %	78,838	20 %	562,860	17 %
	Missing	164,842	39 %	157,408	38 %	139,475	36 %	134,576	35 %	1318,259	39 %

**Fig. 1:**
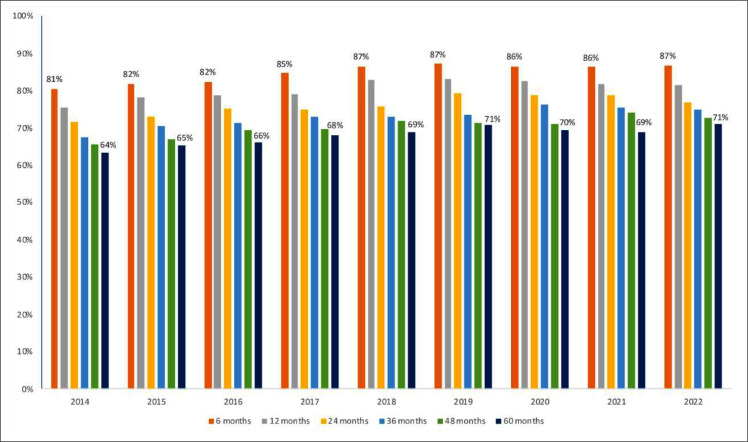
Proportion of people retained on ART, according to time of treatment initiation. Brazil, 2014 to 2022.

The proportion of PLWHA retained to ART have progressively decreased from 6- to 60-months after ART initiation in all years analyzed, reaching 87 % for 6-months versus 71 % for 60-months in 2022. At the same time, an increase was observed from 2014 to 2018 (81 % to 87 % for 6-months vs. 64 % to 69 % for 60-months), with relative stabilization from 2019 to 2022, as shown in Fig. 1.

**Fig. 2:**
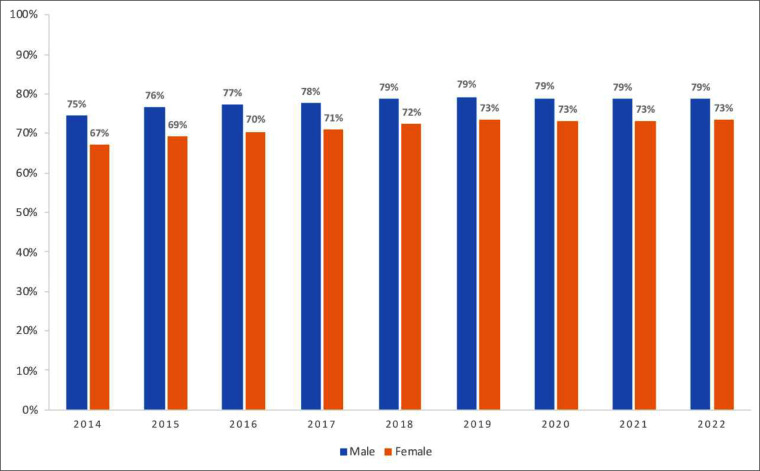
Proportion of people retained on ART, according to sex. Brazil, 2014 to 2022.

Higher proportion of individuals retained on ART were observed among men, ranging from 75 % in 2014 to 79 % in 2022, while in women it was 67 % and 73 %, respectively (Fig. 2).

**Fig. 3:**
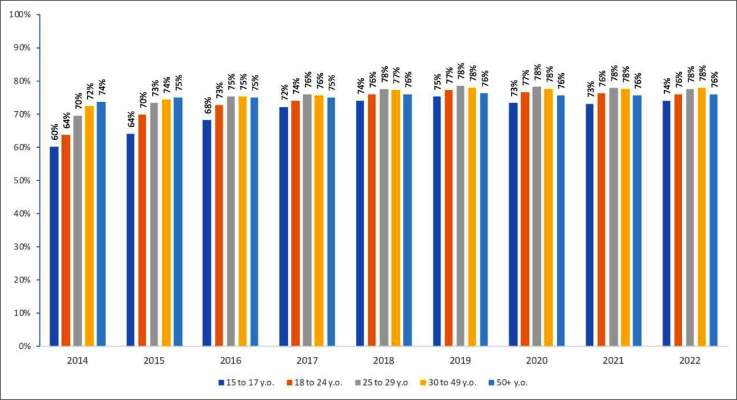
Proportion of people retained on ART, according to age. Brazil, 2014 to 2022.

Between 2014 and 2018, there was an increase in the proportion of people retained on ART in the 15 to 17 (60 % to 74 %) and in the 18 to 24 age group (64 % to 76 %). The proportion of individuals retained remained around 75 % in the 25 to 29-years, 30- to 49-years, and over 50-years age groups throughout the period (Fig. 3).

**Fig. 4:**
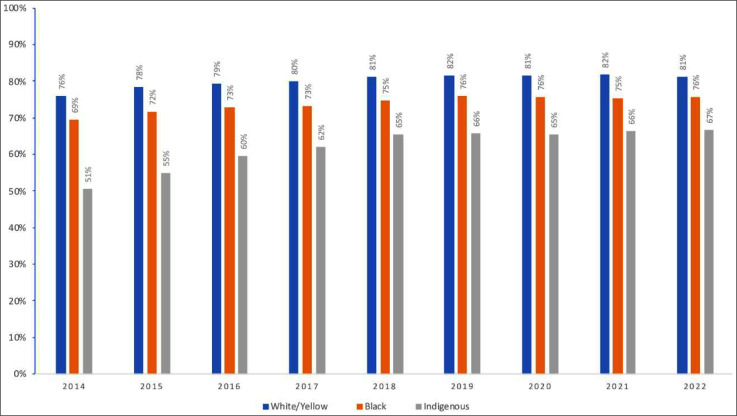
Proportion of people retained on ART, according to race/color. Brazil, 2014 to 2022.

Important differences were observed in relation to race/color, with lower retention levels observed in the indigenous (from 51 % in 2014 to 67 % in 2022) and black population (from 69 % in 2014 to 76 % in 2022), when compared to the white/yellow population (from 76 % in 2014 to 81 % in 2022), throughout the analyzed period (Fig. 4).

**Fig. 5:**
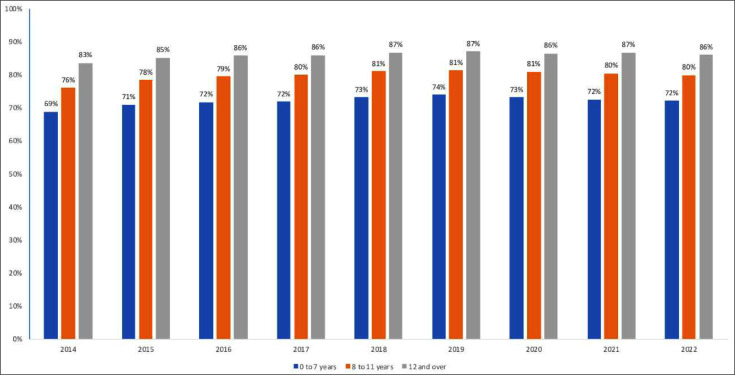
Proportion of people retained on ART, by years of study. Brazil, 2014 to 2022.

Individuals with higher levels of education presented higher levels of retention throughout the period, with 86 % of people retained on ART among those with 12-years of schooling or more, versus 72 % among those with 0- to 7-years of schooling, in 2022. (Fig. 5).

**Fig. 6:**
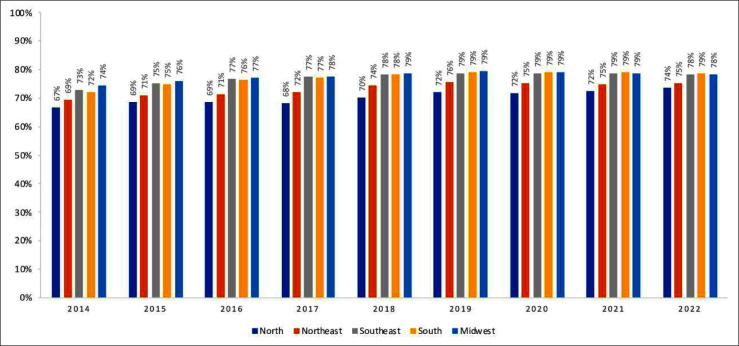
Proportion of people retained on ART, by Region. Brazil, 2014 to 2022.

Some regional differences were observed, with the Southeast, South and Mid-West regions presenting the highest proportions of PLHIV retained to ART (78 %, 79 % and 78 % respectively, in 2022) followed by the Northeast and North regions (74 % and 75 % respectively, in 2022), as shown in Fig. 6.

## Discussion

Our findings revealed a significant and progressive decrease in ART retention over time, reaching concerning levels at 60-months of treatment. This pattern is consistent with international evidence. A systematic review and meta-analysis conducted in low- and middle-income countries demonstrated a similar decline in ART retention over time ‒ 78 %, 71 %, and 69 % at 12-, 24-, and 36-months, respectively ‒ corroborating observations from other studies that maintaining high levels of ART retention over time remains a challenge in many countries.^7^^,^^12^^,^^14^^,^^15^

The persistently low levels of retention at 60-months observed throughout the study period is a matter of concern, given the chronic nature of HIV infection and the requirement for lifelong treatment. Suboptimal retention increases the risk of virologic failure, drug resistance, and HIV-related morbidity and mortality.^6^, ^7^, ^8^, ^9^

This study observed a relative stability of retention rates from 2018 to 2022, while many countries experienced a negative impact of the SARS-CoV-2 pandemic across HIV continuum of care cascade, including retention, adherence, and viral suppression.^16^^,^^17^ In Brazil, this stability may be explained by strategies implemented by the Ministry of Health aimed at mitigating possible negative impacts of the pandemic on HIV care, such as the expansion of multi-month ARV dispensation and the implementation and expansion of telemedicine for the ARV prescription and follow-up of PLWHA.^18^, ^19^, ^20^ The introduction of integrase inhibitor-based regimens in previous years, with fewer side effects and improved tolerability, may also have contributed to the maintenance of retention during this period.^21^, ^22^, ^23^

We also found that men had higher retention rates than women. This finding aligns with studies conducted in Indonesia and Canada, in which women demonstrated lower retention and adherence, potentially due to factors such as social stigma, gender-based barriers to care, lack of family or partner support, and mental health challenges.^24^, ^25^, ^26^

Some studies have identified lower levels of adherence to ART among women, which may be related to factors such as marital approval, stigmatization, and mental health issues.^26^ However, other studies have observed that men were more likely to be lost to follow-up, indicating that gender disparities in ART retention may be context-specific and require further investigation.^27^, ^28^, ^29^

Although progress has been made over time, lower retention levels were observed among the population aged 15- to 17-years, particularly between 2014 and 2016. This finding is consistent with other national and international studies, which have highlighted the unique challenges faced by adolescents in adhering to lifelong treatment regimens.^30^, ^31^, ^32^, ^33^, ^34^, ^35^

Racial and ethnic disparities were also evident in our study. Black and Indigenous population had lower retention rates compared with White and Asian populations. This pattern echoes previous findings in Brazil, where these populations are more likely to experience late HIV diagnosis, poor retention, and unsuppressed viral load.^36^ Structural barriers and inequities in access to healthcare services may contribute to these outcomes.^33^^,^^37^^,^^38^

Geographical disparities were also observed, with the North and Northeast regions presenting the lowest retention rates. Despite Brazil’s long-standing policy of universal access to ART, services remain unevenly distributed, with a greater concentration in urban centers, particularly in the South and Southeast regions.^39^, ^40^, ^41^

Education attainment emerged as an important factor for retention. Individuals with higher levels of education were more likely to remain in treatment, supporting existing evidence that lower education levels are associated with lower adherence rates and retention in ART, as well as higher levels of loss to follow-up.^42^^,^^43^

A methodological strength of this study was the development and application of an approach to assessing ART retention using national-level data from Brazil’s ART dispensation database. This method enabled us to define retention status at various time points after ART initiation while accounting for patients receiving multi-month dispensation. As an opportunity for improvement, the Brazilian Ministry of Health may revise its methods for analyzing ART retention based on the approach used in our study.

Nonetheless, some limitations should be noted. Retention was inferred from pharmacy refill data and may not fully capture true engagement in care, adherence, or virologic suppression. The descriptive nature of this study and the absence of formal statistical testing or trend modeling did not allow us to determine whether the observed changes in retention were attributable to specific programmatic interventions. The inclusion criterion of at least one dispensation may have captured individuals who initiated ART but never meaningfully engaged in care, potentially influencing early retention estimates. We were unable to exclude HIV/AIDS-related deaths during the study period. However, a previous population-based study conducted in Brazil showed that out of the 10 to 12 thousand annual cases of AIDS-related deaths in the country, almost half occurred among PLWHA who had not started ART.^44^ Considering the magnitude of the population analyzed in this study, no considerable impact on results is expected due to not excluding AIDS-related deaths from the analysis. Additionally, there were high levels of missing data for race/ethnicity and education, which may bias subgroup analyses and likely lead to underestimation of disparities; therefore, findings related to these variables should be interpreted with caution. This limitation underscores the importance of strengthening the completeness and quality of health information systems, as accurate and comprehensive sociodemographic data are essential to adequately characterize demographic and clinical profiles, identify inequities, and inform more targeted and effective public health and clinical interventions. Although plausible, the interpretations proposed to explain the observed trends ‒ such as the impact of pandemic mitigation strategies and the introduction of ARV regimens ‒ remain speculative and cannot be formally tested within the design of the present study.

We also used aggregated data and assessed ART retention at fixed time points, which limited our ability to evaluate continuous retention dynamics. As a study based on secondary data, our findings raise important hypotheses but cannot establish causality. Further research is needed to better understand the factors driving retention in the Brazilian context.

By introducing an innovative methodology based on delays in ART dispensation and applying it to a national database, this population-based study contributes new evidence on ART retention trends at multiple time points after ART initiation in Brazil. Our findings build upon previous research on ART adherence and retention, emphasizing the challenges of maintaining long-term ART engagement even in a setting with universal access. Importantly, they reveal persistent disparities in retention among vulnerable populations, underscoring the need for targeted and equitable public health interventions to sustain engagement in HIV care.

## Conclusions

This was the first study to analyze ART retention trends at multiple time points using national-level data in Brazil. We observed a gradual decline in retention over time, with marked disparities across sociodemographic groups. Although retention remained relatively stable during the COVID-19 pandemic, long-term retention remains a key challenge. Our methodology offers a valuable tool for routine monitoring of ART retention and reinforces the need for equity-focused strategies to improve retention. While this study provides important population-level insights, longitudinal cohort studies are needed to assess individual care journeys and better understand the factors influencing sustained engagement in HIV care. Qualitative research may help uncover structural and psychosocial barriers to retention. Expanding differentiated care, strengthening community support, and addressing social determinants of health are critical to sustaining ART engagement and advancing HIV epidemic control.

## Funding

This research did not receive any specific grant from funding agencies in the public, commercial, or not-for-profit sectors.

## Data availability

The data that support the findings of this study are available from the corresponding author upon reasonable request.

## Conflicts of interest

Marcelo A. de Freitas is currently an employee of GlaxoSmithKline (GSK). This study was conceived and initially conducted prior to his employment at GSK, which had no role in the study design, data collection, data analysis, data interpretation, or decision to publish the manuscript. All other authors declare no competing interests.
